# A Systems-Wide Approach for Early Detection and Management of Dementia in Primary Care

**DOI:** 10.1093/geroni/igab046.1335

**Published:** 2021-12-17

**Authors:** Annette Fitzpatrick, Basia Belza

## Abstract

Dementia is frequently unrecognized and under-reported by health care providers. The needs of an aging population increase the burden on an already over-worked primary care system that is often without the appropriate training, resources, and reimbursement to address the growing number of people with cognitive decline in the US. In this symposium we present a systems-wide approach within University of Washington (UW) Primary Care to increase awareness of early signs and symptoms, detection of cognitive impairment, and support of providers, patients and caregivers that will ultimately improve outcomes of care. This quality improvement (QI) program integrates stakeholder-selected components of the GSA KAER (Kickstart-Assess-Evaluate and Refer) Model and Toolkit (2020 Edition), developed by the Gerontological Society of America (GSA), into primary care practice. We describe content and logistics of a continuing education intervention for primary care providers and clinical staff to increase skills for evaluation and management of dementia. Working with UW clinic managers and information technology (IT), we have developed a pragmatic system for streamlining operations and documenting care utilizing newly developed interdisciplinary workflows and electronic health record order sets. Using input from our Community Advisory Board, we explain development of a web-based resource directory to be used in-clinic and at home to support providers, staff, patients, families, and caregivers across cognitive changes. Strategies presented here are aimed to help other health care systems initiate steps to integrate KAER and other tools into a practical QI program for improving detection and management of dementia through support of primary care.

